# What I Know about Phytolacca

**Published:** 1885-04

**Authors:** E. B. Nash

**Affiliations:** Cortland, N. Y.


### WHAT I KNOW ABOUT PHYTOLACCA.

The article by William J. Guernsey under the above title is of the kind that ought much oftener to grace the pages of our journals, because they do more to "help those who are half way," as Father Hering used to say, than anything else that could be offered, for they place the opportunity to "prove whether these things be true," within easy reach of all who are honestly trying to understand and apply pure Homœopathy, and especially the potentized form of it, to the healing of the sick.

When I began the practice of medicine, having studied with a low-dilution alternater, and attended lectures in a college where but one of the whole faculty used or recommended anything above the third or sixth, and *he* never above the thirtieth, I was, of course, not naturally inclined to potencies. Indeed, I was so utterly prejudiced *against* them that I would not have one of them in my office. There was no amount of *argument* that could convince me. In 1866 I took the old *American Homœopathic Review*. It was the only journal I took then, for I could not afford to take more. Of course, I read it, and was led to the experiment with the thirtieth and two hundredth potencies by reading the *cures* made with these potencies by such men as Dunham, Wells, Lippe, Hering, Morgan, Boyce, and others. With all my prejudices, I could not get over two facts, viz.: that their potentized remedies (as reported in their cases) were chosen from *clean-cut indications*, and that they made quicker and more perfect cures than I had been able to do with my low preparations, singly or alternated.

Then I experimented, carefully at first and with little faith, but honestly and thoroughly, and have been experimenting ever since, and so far with this result: If I were compelled to choose one potency with which to treat all diseases, both acute and chronic, I would not go below the *two hundredth.*

Now, I repeat my convictions, that solid facts in the way of cures with the well indicated, potentized remedy, and experience like that in Dr. G.'s article, will do more to lead the young and old in the way of truth than any possible amount of theorizing and controversy. Here let me add my mite to that of Dr. G., and if I do not give any new light, make stronger the old by telling what I know about Phytolacca.

The patient is (with or without a chill) attacked with severe head, back, and limbs ache, and very high fever. This is often imagined to be the beginning of a fever (typhoid), but following close upon these general or constitutional symptoms, the throat begins to be sore. Tonsils swollen, with pains shooting up into one or both ears, especially when trying to swallow. Then little white or yellow spots appear upon the tonsils, which, if the case is a severe one, soon coalesce and form patches of membrane. The breath is putrid and the body is often sore as if bruised (like Arnica), so much so that the patient groans with pain, especially when trying to move or turn over in bed. He also gets sick and dizzy when trying to sit up. No need of naming this case, because naming will not help us to cure the symptoms which call for Phytolacca. We give it in solution, one in two to four hours, according to the violence of the case, until the fever and general pains and aching begin to subside (which is generally within six to twenty-four hours), then withdraw the remedy, to repeat when the improvement seems to flag.

The local manifestations in the throat generally follow the subsidence of the constitutional symptoms within the next twenty-four or forty-eight hours, and the case is cured. I have no hesitation in saying that I have treated hundreds of cases of which this is a picture. Formerly I used four drops of the $\theta$ in a teacup of cold water, dessert-spoonful doses. Latterly I used the thirtieth dil. of my own preparation.

I find Phytolacca one of the best remedies for difficult dentition. Many cases that have obstinately resisted the more commonly used Acon., Bell., Cham., Calc., and Merc. have in my hands been quickly and permanently relieved by the Scoke root. Symptoms: Child crying, moaning, restless, and feverish, particularly at night. If it is hot weather, often vomiting and diarrhœa. The teeth a long time coming, and the crowning characteristic indication is that the child wants to bite on something hard continually, and seems relieved by it. Retarded dentition is often relieved better by Phyto. than by Calc. or Sil. notwithstanding it has not been shown to enter into the composition of bone that I know of, nor is it pre-eminently a tissue remedy according to the Schussler plan.

*Again:* If there is any one remedy that is worthy our fullest confidence in the average scarlatina of our zone it is this one. If any one doubts the homœopathicity of it to this disease let him examine its pathogenesis in Allen, and note the angina, coryza, delirium, fever, general aching, especially in the limbs, and be convinced. Under its use the now-appearing eruption "blossoms like a rose," and the other symptoms, so intense until its appearance, are correspondingly relieved.

And now with its efficacy in quinsy, mastitis, "skyattic," of Guernsey, and those affections of which I have written, what a truly valuable remedy we have in Phytolacca.

Won't the Doctors Guernsey tell us something more of what they know of our indigenous remedies? I believe there are hundreds of physicians all over the land who would with me join in saying, "Well done, good and faithful."

E. B. NASH.

CORTLAND, N. Y.

